# Enhanced electrochemical performance of Lithium-ion batteries by conformal coating of polymer electrolyte

**DOI:** 10.1186/1556-276X-9-544

**Published:** 2014-10-02

**Authors:** Nareerat Plylahan, Sébastien Maria, Trang NT Phan, Manon Letiche, Hervé Martinez, Cécile Courrèges, Philippe Knauth, Thierry Djenizian

**Affiliations:** 1 Aix-Marseille Université, CNRS, LP3 UMR 7341, F-13288, Marseille Cedex 9, France; 2 Aix-Marseille Université, CNRS, MADIREL UMR 7246, F-13397, Marseille Cedex 20, France; 3 Aix-Marseille Université, CNRS, ICR UMR 7273, F-13397, Marseille Cedex 20, France; 4 IPREM-ECP-UMR 5254, Université de Pau et des Pays de l’Adour, Hélioparc Pau-Pyrénées, 2 Av du Président Angot, 64053, Pau Cedex 9, France

**Keywords:** Titania nanotubes, Polymer electrolyte, Electropolymerization, Lithium-ion batteries

## Abstract

This work reports the conformal coating of poly(poly(ethylene glycol) methyl ether methacrylate) (P(MePEGMA)) polymer electrolyte on highly organized titania nanotubes (TiO_2_nts) fabricated by electrochemical anodization of Ti foil. The conformal coating was achieved by electropolymerization using cyclic voltammetry technique. The characterization of the polymer electrolyte by proton nuclear magnetic resonance (^1^H NMR) and size-exclusion chromatography (SEC) shows the formation of short polymer chains, mainly trimers. X-ray photoelectron spectroscopy (XPS) results confirm the presence of the polymer and LiTFSI salt. The galvanostatic tests at 1C show that the performance of the half cell against metallic Li foil is improved by 33% when TiO_2_nts are conformally coated with the polymer electrolyte.

## Background

The miniaturization of Lithium-ion batteries (LIBs) as a power source to drive small devices has been continuously developed to meet the market requirements of nomadic applications. The challenge of the miniaturization of LIBs is to minimize the size and, at the same time, maximize the energy and power densities of the battery. In this context, 3D Li-ion microbatteries have been developed to overcome this challenge. Particularly, nanoarchitectured electrodes such as self-organized titania nanotubes (TiO_2_nts) are a potential candidate as a negative electrode in 3D Li-ion microbatteries [1-9]. TiO_2_nts show a better electrochemical performance compared to the planar TiO_2_ counterpart [6] due to i) their high active surface area, ii) the direct contact between the active material and the substrate, iii) fast diffusion of charges, and iv) high stability upon cycling owing to spaces between nanotubes, which allow the volume variation caused by Li^+^ insertion/extraction. To achieve the fabrication of the full 3D Li-ion microbatteries, the use of conventional organic liquid electrolytes must be avoided due to the safety concerns and its incompatibility to the integrated circuit technology. Poly(ethylene oxide) or poly(ethylene glycol) (PEG) is the most common polymer electrolyte used in LIBs due to its compatibility to the all-solid-state Li-ion microbatteries [10], promising ionic conductivity [11,12], and thermal stability [13]. However, it is necessary to maintain the 3D nanotubular structure of TiO_2_nts after the deposition of the polymer electrolyte in order to subsequently fabricate the full 3D Li-ion microbatteries by filling the polymer-coated TiO_2_nts with a cathode material. Recently, we have reported the electropolymerization by cyclic voltammetry (CV) to conformally electrodeposit the PEG-based polymer electrolyte with bis(trifluoromethanesulfone)imide or LiTFSI salt on the TiO_2_nts without closing the tube opening [14-16]. The conformal electrodeposition of polymer is a convenient approach to preserve the 3D morphology of the substrate as it has been reported for other materials [17]. The main advantage of the conformal coating is that all the active surface area of TiO_2_nts is in contact with the polymer electrolytes, which will improve the charge transport and thereby improving the performance of the microbatteries. It can be noted that the electrochemical-assisted polymerization process is a convenient and versatile approach. Indeed, the polymer formation can be achieved by different mechanisms involving the formation of free-radical intermediates, the activation by light, the use of initiator, and the direct oxidation or reduction of monomer as it has been reported for poly(para-phenylene)vinylene (PPV) [18], polypyrrole (PPy) [19], poly(2-methacryloyloxy(ethyl) acetoacetate) [20], and poly(sulfonated phenol) [21], respectively.

In this work, we report the fabrication of highly ordered and smooth TiO_2_nts and the conformal coating of the polymer electrolyte: comb-shaped poly(poly(ethylene glycol) methyl ether methacrylate) or P(MePEGMA) on TiO_2_nts by an electropolymerization technique. The morphology of the materials was characterized by scanning electron microscopy (SEM) and transmission electron microscopy (TEM). The chemical structure of the obtained P(MePEGMA) polymer was analyzed by proton nuclear magnetic resonance (^1^H NMR) and its molar mass was measured by size-exclusion chromatography (SEC). The polymer electrolyte was studied in depth by X-ray photoelectron spectroscopy (XPS). The electrochemical tests were carried out in the half cell to investigate the improved performance of the solid-state batteries caused by the conformal coating of TiO_2_nts with the polymer electrolyte.

## Methods

TiO_2_nts were synthesized by electrochemical anodization [22] of Ti foil in an electrolyte containing 96.7 wt% glycerol, 1.3 wt% NH_4_F, and 2 wt% water. A constant voltage of 60 V was applied to the cell (Ti foil as a working electrode and Pt foil as a counter electrode) for 3 h. After the anodization, the sample was rinsed with deionized water without removing it from the cell.

The electropolymerization on as-formed TiO_2_nts was carried out by CV [14-16] in an aqueous electrolyte containing 0.5 M LiTFSI and 0.2 M poly(ethylene glycol) methyl ether methacrylate or MePEGMA in a three-electrode system: TiO_2_nts as a working electrode, Pt foil as a counter electrode, and Ag/AgCl, 3 M KCl as a reference electrode. MePEGMA is used as a starting monomer with an average molecular weight of 300 g mol^-1^ and an average repeating ethylene oxide unit of 5. Prior to the electropolymerization, the electrolyte was purged with N_2_ for 10 min to remove dissolved oxygen. The CV was done for five cycles in the three-electrode configuration at the scan rate of 10 mV/s, potential window of -0.35 to -1 V vs Ag/AgCl, 3 M KCl. After the electropolymerization, the sample was dried without rinsing under vacuum at 60°C overnight.

The morphology of TiO_2_nts and polymer-coated TiO_2_nts were examined by electron microscopy techniques using a JEOL 6320 F SEM and a JEOL 2010 F TEM (JEOL Ltd., Akishima, Tokyo, Japan).

^1^H NMR spectra in CDCl_3_ were recorded on a Bruker Advance 400 spectrometer (Bruker AXS, Inc., Madison, WI, USA). A chemical shift was given in parts per million relative to tetramethylsilane. In order to characterize the polymer by NMR, the sample was immersed during approximately 1 h in CDCl_3_. Then the solution was recovered and concentrated to reach the minimum volume for an NMR analysis (≈0.5 mL).

Polymer molecular weights and dispersities were determined by SEC. The used system was an EcoSEC (Tosoh, Tokyo, Japan) equipped with a PL Resipore Precolumn (4.6 × 50 mm, Agilent Technologies, Inc., Santa Clara, CA, USA) and two linear M columns (4.6 × 250 mm, Agilent) with a gel particle diameter of 3 μm. These columns were thermostated at 40°C. Detection was made by an UV/visible detector operated at *λ* = 254 nm, a dual flow differential refractive index detector, both from Tosoh, and a viscometer ETA2010 from PSS (Polymer Standards Service Inc., Amherst, MA, USA). Measurements were performed in THF at a flow rate of 0.3 mL min^-1^. Calibration was based on polystyrene standards from Polymer Laboratories (ranging from 370 to 371,100 g mol^-1^).

XPS measurements were performed on a Thermo K-alpha spectrometer (Thermo Fisher Scientific, Waltham, MA, USA) with a hemispherical analyzer and a microfocused monochromatized radiation (Al Kα, 1,486.7 eV) operating at 72 W under a residual pressure of 1 × 10^-9^ mbar. Peaks were recorded with a constant pass energy of 20 eV. The spectrometer was calibrated using the photoemission lines of gold (Au 4f7/2 = 83.9 eV, with reference to the Fermi level) and copper (Cu 2p3/2 = 932.5 eV). The Au 4f7/2 full width at a half maximum (FWHM) was 0.86 eV. All samples were fixed on the sample holders in a glove box directly connected to the introduction chamber of the spectrometer to avoid moisture/air exposure of the samples. Charge effects were compensated by the use of a charge neutralization system (low-energy electrons) which has the unique ability to provide consistent charge compensation. Short acquisition time spectra were recorded before and after each experiment and compared, to check the non-degradation of the samples under the X-ray beam. The binding energy (BE) scale was calibrated from the hydrocarbon contamination using the C 1 s peak at 285.0 eV. Core peaks were analyzed using a linear background, except for the S 2p core peak for which a non-linear Shirley-type background was applied [23]. The peak positions and areas were obtained by a weighted least-square fitting of model curves (using 70% Gaussian and 30% Lorentzian line shapes) to the experimental data. Quantification was performed on the basis of Scofield's relative sensitivity factors [24].

The electrochemical tests of bare TiO_2_nts and polymer-coated TiO_2_nts were carried out using galvanostatic measurements. TiO_2_nt electrodes were assembled against a metallic Li foil using Swagelok cells in an Ar-filled glove box. Two sheets of Whatman paper soaked with MePEGMA + LiTFSI electrolyte were used as a separator. The separators were prepared by soaking the circular disk (diameter of 10 mm) with an aqueous solution of 0.5 M LiTFSI + 0.8 M MePEGMA, then dried at 60°C in a vacuum dryer overnight. The cells were cycled at 1C in the potential window of 1.4 to 3 V vs Li/Li^+^ using VMP3 (Biologic instrument).

## Results and discussion

**Figure 1:**
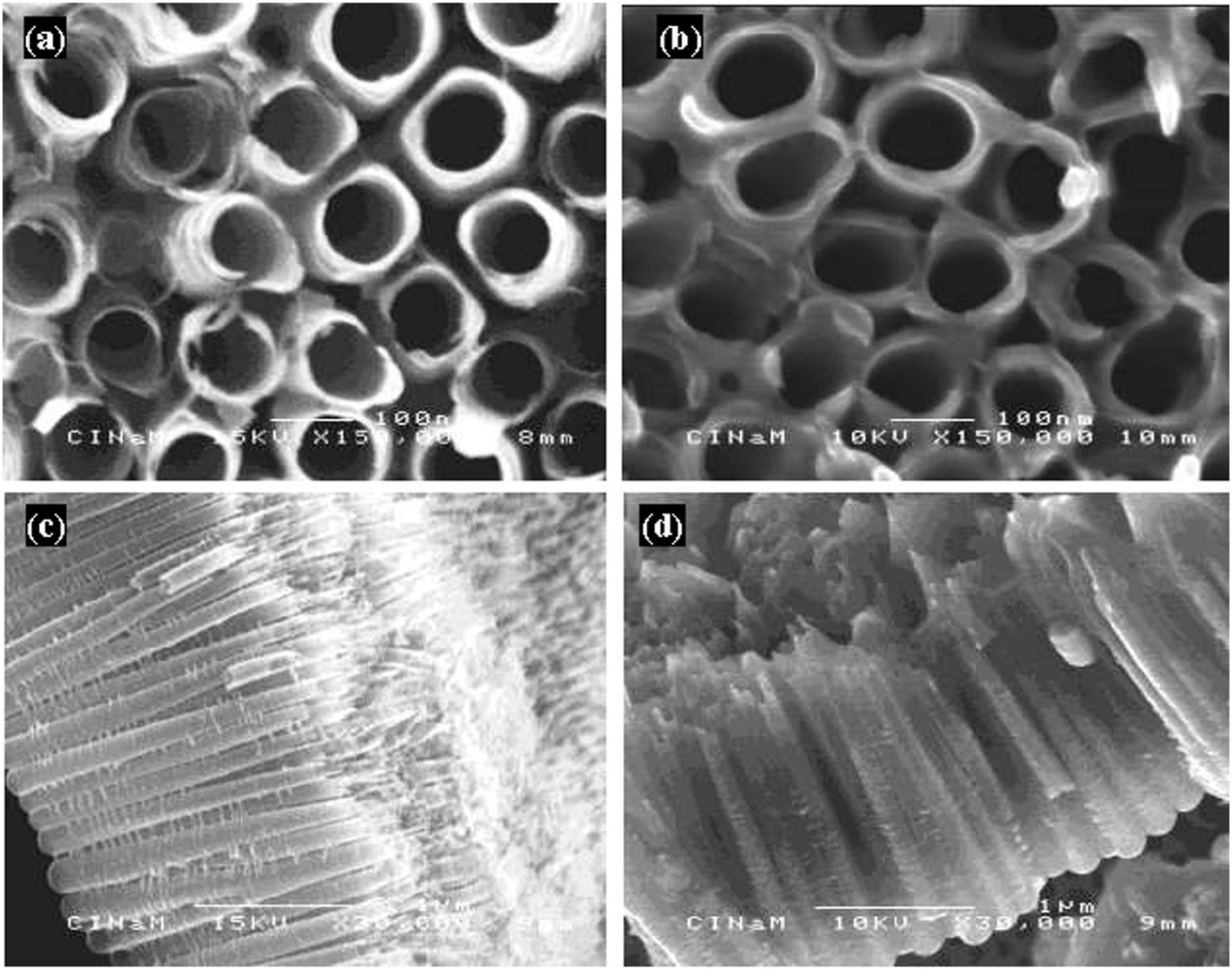
**Morphology of as-formed TiO**_**2**_**nts and polymer-coated TiO**_**2**_**nts.** SEM images of **(a)** top view of as-formed TiO_2_nts, **(b)** top view of polymer-coated TiO_2_nts, **(c)** cross section of as-formed TiO_2_nts, and **(d)** cross -section of polymer-coated TiO_2_nts.

The morphology of as-formed TiO_2_nts and polymer-coated TiO_2_nts is shown in Figure 1. In Figure 1a,c, it can be seen that highly organized TiO_2_nts are obtained after the anodization for 3 h. The top view (Figure 1a) shows the rounded shape of the tube opening with the diameter of approximately 100 nm and the wall thickness of 10 nm. The side view (Figure 1c) shows length of the tubes of approximately 1.5 μm. Figure 1b,d shows the TiO_2_nts after the electropolymerization. The conformal electrodeposition of the polymer electrolyte is supposed to occur according to a mechanism involving the formation of hydrogen free radicals as it is described elsewhere [15,25]. The top view (Figure 1b) shows the thicker tube wall of approximately 20 nm which results from the deposition of the polymer. The side view (Figure 1d) shows that the spaces between tubes are filled with the polymer.

**Figure 2:**
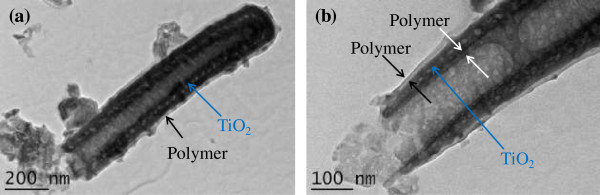
**TEM images of a single tube from the polymer-coated TiO**_**2**_**nts sample.** TEM images of **(a)** a single tube with conformal polymer coating and **(b)** enlarged view at the open end of the polymer-coated tube.

Figure 2 shows the TEM images of a single tube from the polymer-coated TiO_2_nts sample. In Figure 2a, a thin layer of polymer is clearly observed on the outer wall, from the top to the bottom of the tube. The enlarged view at the open end of the tube given in Figure 2b shows that the thickness of the conformal polymer layer is around 10 nm.

**Figure 3:**
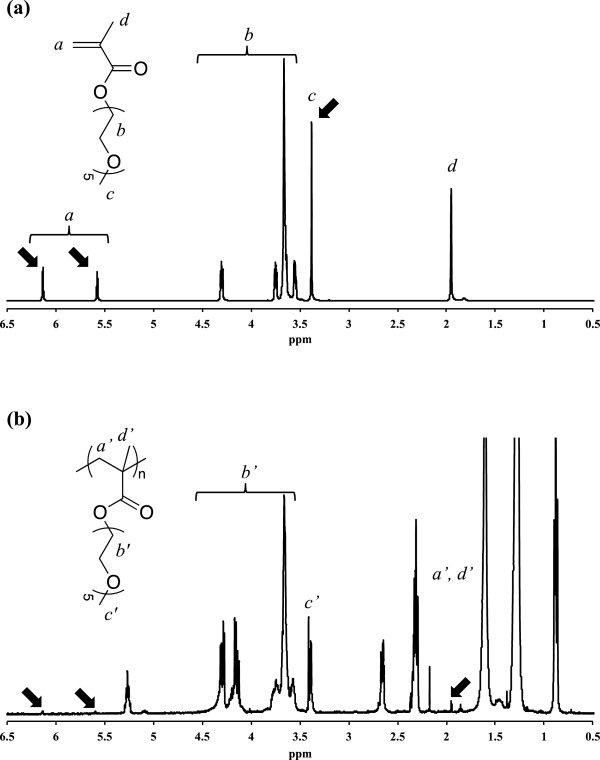
^**1**^**H NMR spectra of (a) MePEGMA monomer and (b) P(MePEGMA) polymer.**

After the conformal deposition of electrolyte on TiO_2_nts has been achieved, the polymer electrolyte was recovered and characterized by ^1^H NMR to verify if the polymer is formed and by SEC to estimate chain length of the polymer. The ^1^H NMR spectra of the MePEGMA monomer and the P(MePEGMA) polymer are shown in Figure 3a,b, respectively. First, the polymer was successfully extracted since the peak at 3.65 ppm corresponding to the methylene protons of the PEG chains together with the peak at 3.40 ppm of the methyl ether end-chain are still present in the polymer spectrum. Moreover the electropolymerization occurred since the peaks at 6.11 and 5.58 ppm corresponding to the vinylic protons and the singlet at 1.95 ppm of the methyl of the unsaturated methacrylic units in the monomer spectrum almost disappeared in the spectrum of the polymer (see arrows in Figure 3). Additionally new peaks appeared in the polymer spectrum between 2.8 and 0.8 ppm, which are unfortunately not easy to assign. This area of the spectrum should contain peaks corresponding to the methylene and methyl groups of the methacrylate backbone but also some impurities. Indeed, considering the small volume taken by the nanostructured polymer layer in the sample, the overall quantity of polymer available is very low and it was difficult to avoid pollution of the extracted material. For example, peaks at 0.88 and 1.27 ppm could indicate the presence of linear alkanes and singlet at 1.6 ppm is due to water.

**Figure 4:**
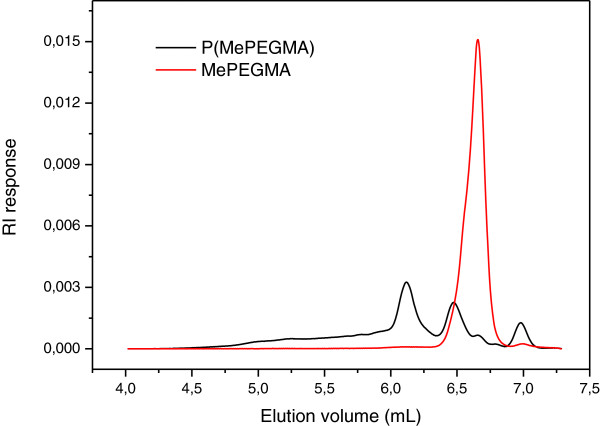
SEC chromatograms of P(MePEGMA) and MePEGMA.

The SEC chromatograms of P(MePEGMA) polymer and MePEGMA monomer are shown in Figure 4. There are three evident peaks at the elution volume of 6.15, 6.5, and 7 mL. It is noted that the average molecular weight of the monomer used in this work is approximately 300 g mol^-1^. Hence, the peak at 6.5 mL is nearly superimposed to the peak corresponding to the monomer; this means that our electrolyte contains probably the residual monomer, with modified end-chains considering the NMR results. The peak at 6.15 mL shows the highest intensity (800 g mol^-1^), which is three times the molecular weight of the monomer peak at 6.5 mL. Therefore, this peak can be assigned to trimers. The peak at 7 mL with the molecular weight of 154 g mol^-1^ is lower than the weight of the monomer. This peak may correspond to the broken side chain from the monomer or trimers. We also observe a very large shoulder between 5 and 6 mL indicating the formation of larger polymer species, but the signal intensity is too low to get molar mass integration.

XPS measurements were performed on self-organized TiO_2_nts coated with P(MePEGMA) and TiO_2_nts coated with P(MePEGMA) + LiTFSI. All the quantitative data, including binding energies of the different species detected, full width at maximum height (FWMH) and atomic percentages (At.%) are given in Table 1. For TiO_2_nts + P(MePEGMA) sample, O 1 s and C 1 s peaks are detected. These peaks mainly result from the oxygen and carbon atoms in the polymer. The F 1 s peak is attributed to the residual fluoride ions from the anodization process since the anodization and the electropolymerization were carried out in the same electrochemical cell. For TiO_2_nts + P(MePEGMA) + LiTFSI sample, N 1 s, F 1 s, Li 1 s, and S 2p peaks are detected in addition to O 1 s and C 1 s peaks, which results from the presence of LiTFSI salt [LiN(SO_2_CF_3_)_2_]. It can be noticed that no Ti and O atoms from TiO_2_nts have been observed in both samples, which indicates that the corresponding nanotubes are completely coated by the polymer electrolyte with a layer thickness over 5 nm (≈depth analysis of XPS experiments), which is in agreement with the thickness determined by TEM analysis.

**Table 1:** **Binding energies (BE, eV), full width at a half maximum (FWHM, %), and atomic percentages (At.%) of the main components of TiO**_**2**_**nts coated with P(MePEGMA) and TiO**_**2**_**nts coated with P(MePEGMA) + LiTFSI**

**TiO**_**2**_**nts + P(MePEGMA)**				**TiO**_**2**_**nts + P(MePEGMA) + LiTFSI**			
	**BE (eV)**	**FWHM (%)**	**At.%**		**BE (eV)**	**FWHM (%)**	**At.%**
C 1 s	285	1.2	11.3	C 1 s	285	1.5	10
	286.5	1.3	44.7		286.5	1.5	17.2
	288	1.1	2.6		288	1.5	2.8
	289.1	1.1	5.2		289.3	1.5	1.9
					292.9	1.2	1.8
O 1 s	531	1.1	0.5	O 1 s	531.4	1.5	2.2
	532.8	1.4	31		532.7	1.6	13.1
	534	1.4	4.1		533.7	1.6	2.2
F 1 s	687.9	2.5	0.6	F 1 s	684.7	1.7	22.6
					688.3	2.3	7
				Li 1 s	55.9	1.4	15.1
				N 1 s	399.6	2.1	1.7
				S 2p	168.9	1.6	2.6
					170.1	1.6	

**Figure 5:**
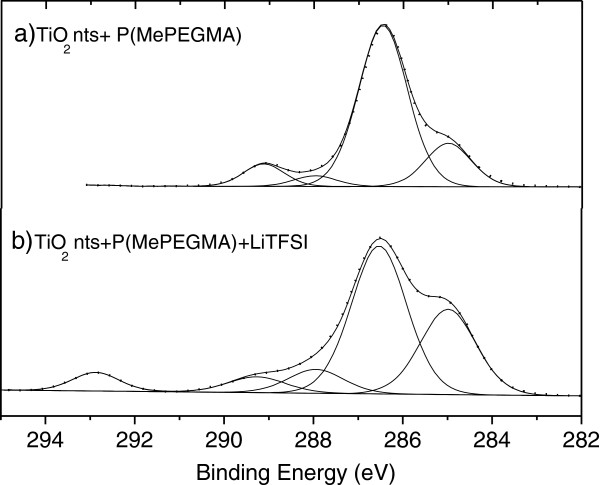
**C 1 s XPS spectra of TiO**_**2**_**nts coated with (a) P(MePEGMA) and (b) P(MePEGMA) + LiTFSI by electropolymerization.**

The XPS spectra of C 1 s core peak for both samples are presented in Figure 5 (for a non-exhaustive presentation, the other XPS spectra are not displayed). The C 1 s core peak of TiO_2_nts + P(MePEGMA) (Figure 5a) presents four main components, which can be assigned to C-C and C-H at 285 eV, C-O at 286.5 eV, O-C-O at 288 eV, and O-C = O at 289.1 eV. The total concentration of C 1 s is 63.9 At.%. The main component located at 286.5 eV (44.7 At.%) corresponds to C-O bond in oligomer species of PEG (-CH_2_-CH_2_-O-)_n_[26]. For C 1 s core peak of TiO_2_nts + P(MePEGMA) + LiTFSI (Figure 5b), the four similar components are observed, plus an additional component at 292.9 eV (1.8 At.%), which can be assigned to CF_3_-like carbon atoms in LiTFSI (LiN(SO_2_CF_3_)_2_), as previously observed by Leroy et al. [27]. The total concentration of C 1 s is 33.6 At.%. The O 1 s core peak of TiO_2_nts + P(MePEGMA) sample is composed of two main peaks at 532.8 eV (31 At.%) and 534 eV (4.1 At.%), which correspond to oxygen in PEG oligomer species. The total concentration of O 1 s is 35.5 At.%. The small component at 531 eV (0.5 At.%) can be attributed to residual OH species on the surface. For TiO_2_nts + P(MePEGMA) + LiTFSI, three components can be assigned for O 1 s peak at 531.2 eV (O-H, 2.2 At.%), 532.3 eV (13.1 At.%), and 533.4 eV (2.2 At.%) (oxygen in PEG and LiTFSI). The O-H bond observed in this sample may result from the adsorbed water by LiTFSI salt on the surface of the sample. The total concentration of O 1 s is 17.7 At.%. It can be noticed that the percentages of C 1 s (286.5 eV) and O 1 s (532.8 and 534 eV) components, relative to C-O (PEG), decrease from TiO_2_nts + P(MePEGMA) to TiO_2_nts + P(MePEGMA) + LiTFSI, which proves the presence of the lithium salt at the surface of TiO_2_nts + P(MePEGMA). The presence of LiTFSI in TiO_2_nts + P(MePEGMA) + LiTFSI sample is confirmed by the XPS spectra of F 1 s (29.3% total atomic percentage), S 2p (2.6% total atomic percentage), Li 1 s (15.1% total atomic percentage), and N 1 s (1.7% total atomic percentage). For F 1 s spectrum, two components can be observed at 684.7 eV (22.6 At.%), which may be assigned to LiF, and 688.3 eV (7 At.%), which corresponds to F-C in LiTFSI. The component of LiF may result from the residual F¯ from the anodization and Li^+^ from LiTFSI salt. It has to be noticed that the proportion between the C 1 s signal relative to C-F (1.8 At.%) and the F 1 s signal of F-C (7 At.%) is in agreement with C-F_3_ ratio in LiTFSI. The peaks of Li 1 s and N 1 s are located at 55.9 eV and 399.6 eV, respectively, and the S 2p signal is composed of S 2p_1/2_ at 168.9 eV and S 2p_3/2_ at 170.1 eV. These results are in agreement with previous XPS core peak characterization of LiTFSI done by Leroy et al. [27] and Plylahan et al. [14].

To summarize, our results show that self-organized TiO_2_nts are successfully coated with the polymer electrolyte, and the presence of PEG and LiTFSI is confirmed.

**Figure 6:**
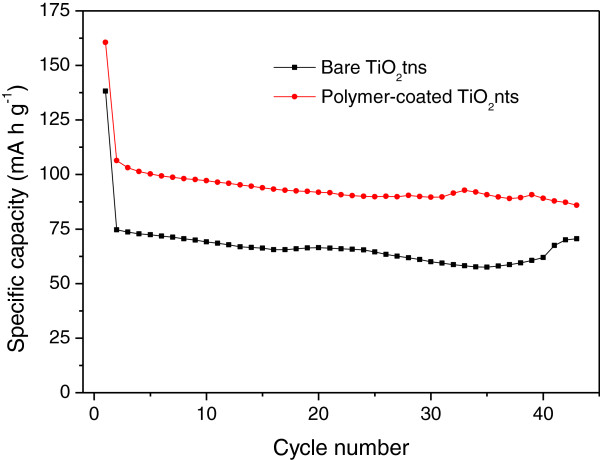
**Specific capacity vs cycle number of bare TiO**_**2**_**nts and polymer-coated TiO**_**2**_**nts at 1C between 1.4 to 3 V vs Li/Li**^**+**^**.**

The positive effect of the conformal coating of the polymer electrolyte on the electrochemical performance of the nanotubes was investigated by galvanostatic tests. Bare TiO_2_nts and polymer-coated TiO_2_nts were tested in a half cell against a metallic Li foil using separator soaked with 0.5 M LiTFSI + 0.8 M MePEGMA. The cycling performance at 1C in the potential window between 1.4 and 3 V vs Li/Li^+^ is shown in Figure 6. The polymer-coated TiO_2_nts deliver an average reversible capacity of 100 mA h g^-1^ which is improved by 33% as compared to bare TiO_2_nts (approximately 75 mA h g^-1^). The better performance is attributed to the conformal coating of the polymer electrolyte that increases the contact area between electrode and electrolyte. This increased contact leads to a better Li^+^ transport and thereby improving the performance of the battery. The irreversible capacity at the first cycle is a normal behavior observed for anatase and amorphous TiO_2_nts [6,9,10,16]. This irreversible capacity is caused by the side reaction of lithium ions and residual water in the polymer and adsorbed surface water on TiO_2_nts. Also, some lithium ions are trapped inside the lattice after the first insertion (first discharge). The capacity fading is fairly low as a result of the 3D nanotubular structure that allows the volume variation during cycling.

## Conclusions

The highly conformal electrodeposition of P(MePEGMA) polymer electrolyte on highly organized TiO_2_nts has been achieved using the cyclic voltammetry technique. The characterization of the polymer electrolyte by ^1^H NMR and SEC shows the formation of short polymer chains, mainly trimers and some traces of high molar mass polymer species as well as MePEGMA monomers. The in depth studies of polymer-coated TiO_2_nts by XPS confirm the presence of polymer and LiTFSI salt. The electrochemical tests of TiO_2_nts in the half cell against metallic Li foil show the improved performance of the LIBs by 33% at 1C when TiO_2_nts are conformally coated with the polymer electrolyte.

## Competing interests

The authors declare that they have no competing interests.

## Authors’ contributions

NP and ML prepared the samples. NP conducted the electrochemical tests and prepared the manuscript. SM and TP conducted ^1^H NMR and SEC experiments and prepared the manuscript. HM and CC conducted the XPS measurements and prepared the manuscript. PK and TD supervised the study. All the authors read and approved the final manuscript.
